# Biobank participants’ perspectives on receiving genetic risk information from a biobank – the case of haemochromatosis

**DOI:** 10.1186/s12920-025-02285-3

**Published:** 2025-12-17

**Authors:** Jonna Clancy, Janina Forstén, Elina Koskinen, Mikko Arvas, Fredrik Åberg, Kimmo Pitkänen, Johanna Castrén

**Affiliations:** 1 Finnish Red Cross Blood Service Biobank, Härkälenkki 13, Vantaa, 01730 Finland; 2 Finnish Red Cross Blood Service, Härkälenkki 13, Vantaa, 01730 Finland; 3 Finnish Red Cross Blood Service R&D, Biomedicum 1, Haartmaninkatu 8, Helsinki, 00290 Finland; 4 Transplantation and Liver Surgery, Helsinki University Hospital and University of Helsinki, Helsinki, Finland

**Keywords:** *HFE* C282Y, Haemochromatosis, Biobank, Blood donor, Genetic information, Actionable genotype

## Abstract

In the era of genomic medicine, utilizing genetic information in the concept of personalized medicine has become widely attractive. In addition to the large-scale population level data sets, professional, standardized and legislated operating environment of the biobanks has enabled their growing footprint in the field of personalized medicine. Moreover, the biobank participants in the Finnish Red Cross Blood Service (FRCBS) Biobank have expressed high willingness to receive information relevant to their health. In this study we screened the FRCBS Biobank genome data, *N* = 43,868, for *HFE* C282Y (+/+) genotype. Clinically verified results were returned to 82 biobank participants (0.19% of the total cohort). In addition, we conducted a survey on their experience on receiving genetic risk information from a biobank. We demonstrate a high occurrence of blood donors not being aware of their genetic risk, a relatively high penetrance of the *HFE* C282Y (+/+) and a clear acceptance of receiving genetic risk information from the biobank by the participants. We show how genetic information stored in a biobank can be used in a precisely defined context, such as blood donation. Further comprehensive studies are needed to fully understand the possibilities biobanks could offer in personalized medicine.

**Supplementary Information:**

The online version contains supplementary material available at 10.1186/s12920-025-02285-3.

## Introduction

Precision medicine has become attractive in past years, due to its potential in enabling more efficient treatment, increasing the quality of life and providing significant cost efficiency [1]. Genetic based precision medicine is widely used especially in the fields of pharmacogenomics and oncology. Several countries have established national genome projects to enhance the usage of genome data to promote health [2], including Finland [3]. Large population-based biobanks in a professional, standardized and legislated operating environment, can serve as an excellent basis for piloting and implementing the use of genetic information in personalized health care [4, 5]. Prerequisites for returning genetic information to sample donors are evidence-based scientific results. Only genetic variants for which the potential morbidity penetrance threshold is met and for which efficacious intervention measures exist should be returned [6, 7].

According to the Finnish Biobank Act, biobank participants’ have the right, upon request, to receive information about health-related factors determined from their biobank sample [8, 9]. In addition, when consenting for biobank in Finland, the sample donors are also asked for a consent to receive health-relevant information. Of the biobank participants in the Finnish Red Cross Blood Service, >99% have consented for receiving health-related information. Real life examples of actively returning genetic information from biobanks exist; genetic risk information related to several disease-associated genetic variants such as ovarian and breast cancer [10] and familial hypercholesterolemia [11] have been returned from the Estonian biobank [5]. In Finland, breast cancer related genetic risk information has been returned to sample donors from all hospital biobanks (Pehrsson et al. 2025, manuscript submitted). To date, the European Society of Human Genetics (ESHG) recommends screening of actionable genotypes to be performed as pilot studies rather than routine screening to gain an understanding on several uncertainties [12], while the American College of Medical Genetics (ACMG) recommends active screening on several identified genetic variants in along with clinical exome sequencing in health care [7].

Hereditary haemochromatosis is an iron accumulation disease caused by mainly founder mutations C282Y and H63D in the *HFE* gene [13, 14] that are common in Northern European populations [15]. According to ACMG recommendation, *HFE* C282Y (+/+) is as an actionable finding as the potential morbidity meets the penetrance threshold and efficacious interventions exist [7]. *HFE* C282Y-H63D compound heterozygotes have been reported to have mildly elevated risk especially together with other predisposing factors [16]. In this study, the screening was restricted to the current ACMG recommendation regarding the mutations in the *HFE* gene. Despite the ACMG recommendation, the low penetrance of the *HFE* C282Y variant raises questions. In Finland, blood donation is regulated by the Blood Service Act, according to which the donation must be voluntary and unpaid [17]. Blood Service Biobank donors represent a regular, and active blood donor population [18]. Post donation iron supplementation is supplied for all frequently donating blood donors, and all women under 50 years of age, to help replenish the iron deficit caused by the blood loss. Individuals with clinical haemochromatosis can donate blood according to the general eligibility criteria for voluntary blood donation if their iron levels are in balance. Therapeutic frequent venesections or other clinical disease monitoring are not carried out in the Blood Service. However, individuals with *HFE* C282Y (+/+) genotype probably should avoid iron supplementation in this context [19]. In this work, we identified biobank participants with genetic predisposition for haemochromatosis, informed them about the finding and provided a recommendation to seek further advice from health care with information enclosed for the health care professional. The aim was to improve blood donor health by not providing futile and potentially harmful iron supplementation in future donations for donors with *HFE* C282Y (+/+) genotype. Furthermore, we conducted a survey to understand the biobank participants’ perspectives of receiving genetic risk information and if the primary finding resulted in clinical endpoints, such as a clinical haemochromatosis diagnosis (ICD10: E83.1).

## Materials and methods

### Identification of *HFE* C282Y homozygotes

The Blood Service Biobank data, *N* = 43,868, previously produced in the FinnGen project, was screened for *HFE* C282Y (+/+) (NM_000410.4:c.845G >A, rs1800562). In brief, the biobank samples were genotyped as part of the FinnGen project with the FinnGen ThermoFisher Axiom custom array v1 or v2. Genotyping, quality control, and genome imputation protocols, R11, are described in detail in FinnGen Gitbook [20]. Shortly, genotype calling was performed with AxiomGT1 algorithm. Prior the imputation, genotyped samples were pre-phased with Eagle 2.3.5 with the default parameters, except the number of conditioning haplotypes was set to 20,000. Genotype imputation was performed using the population-specific imputation reference panel SISu v3 including 3775 high coverage (25–30x) whole genome sequence data, with Beagle 4.1 (version 08Jun17.d8b).

Genotypes were called from variant call format, VFC, files under the following conditions: when imputed dosage score was ≥ 0 and ≤ 0.1, dosage value was considered as 0, when imputed dosage score was ≥ 0.9 and ≤ 1.1, dosage value was considered as 1 and finally when imputed dosage score was ≥ 1.9 and ≤ 2.0, dosage value was considered as 2. Genotype assignments outside of these ranges were set to *missing*. *HFE* C282Y (+/+) screening was performed in R [21] version 3.6.1 or later, with R Studio [22].

### Clinical validation of the preliminary finding

The preliminary *HFE* C282Y (+/+) genotypes were validated in an external EAK (Estonian Accreditation Centre, EA MLA member) accredited laboratory (EVS-EN ISO 15189) in SynLab Tallinn, SynLab (synlab.fi). The clinical grade confirmation of *HFE* C282Y (+/+) genotype was performed with accredited Taqman based genotyping method (B -Hemok-D KL 1858).

### Informing biobank participants

Biobank participants with confirmed *HFE* C282Y (+/+) genotype were contacted by letter. The letter provided information about haemochromatosis, a recommendation to contact their healthcare provider for further information and information about haemochromatosis for their physician. A recommendation that the following laboratory tests should be measured by the healthcare provider was included in the letter; basic blood count, ferritin, transferrin saturation, alanine aminotransferase and, due to the nature of biobank samples as research samples, a confirmatory clinical sample taken for validating the *HFE* C282Y (+/+) genotype. The participants were given the possibility to contact a physician at The Finnish Red Cross Blood Service for any questions related to the information they had received.

Along with the information letter, the biobank participants were asked to return a feedback form answering three questions: (1) Were you aware of your genetic predisposition to haemochromatosis?, (2) Can the finding be recorded to the blood donation registry to avoid the iron supplementation in future blood donations? and (3) Are you willing to take part in a survey about receiving genetic risk information from biobank?

### Survey

A survey was sent by post to all those biobank participants, who agreed in the feedback form to participate in the survey, with an informed consent enclosed. The survey consisted of a total of 26 questions, which covered the participants’ experiences on receiving genetic risk information from the biobank, participants’ experiences on how their matter was handled among the health care providers, participants’ perspectives on the impact of the information received has on their lifestyle behavior and participants’ experiences on sharing the information with first degree family members. The survey form is available in the Supplementary File 1. The survey was administered in Finnish or in Swedish and then translated into English for publishing purposes.

## Results

**Fig. 1:**
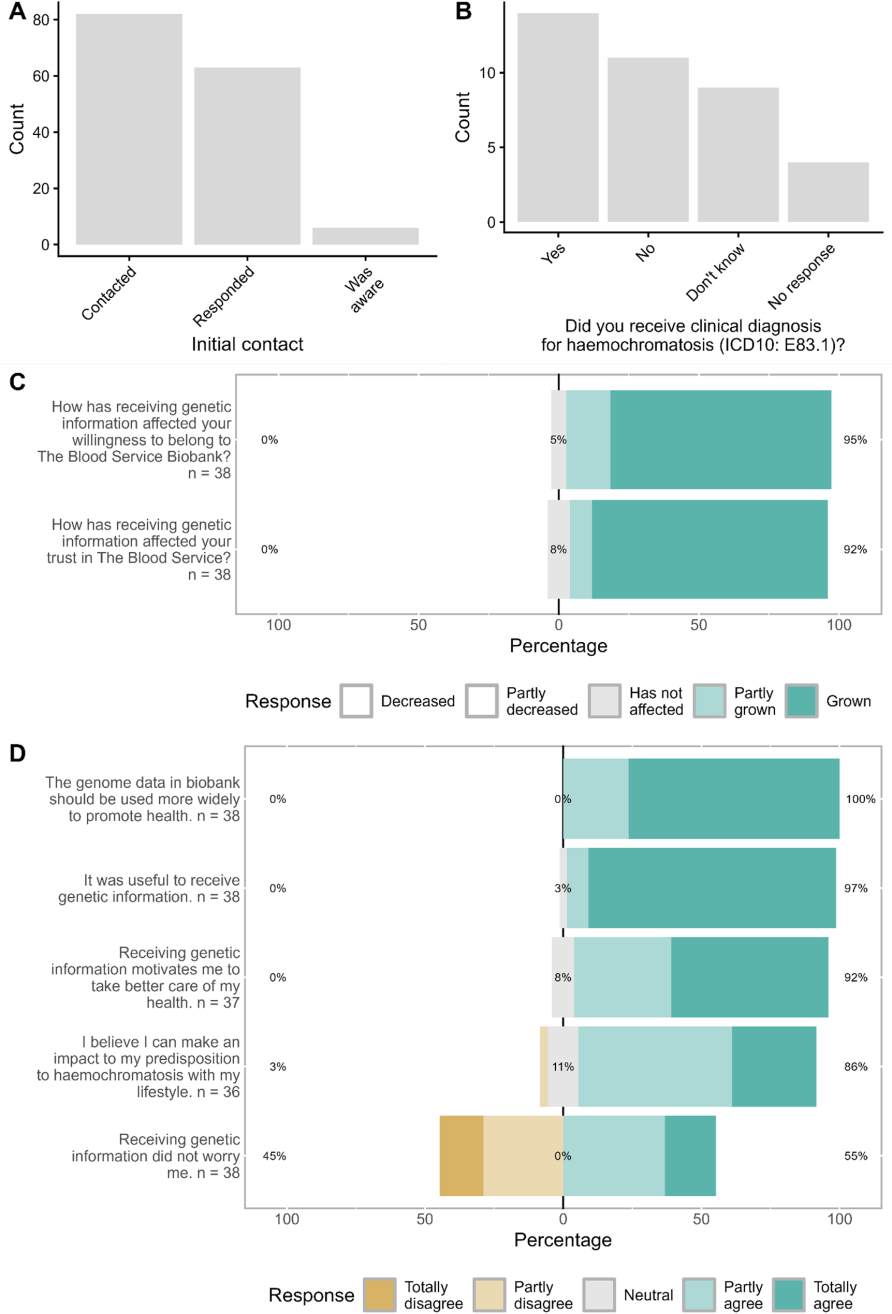
Participants’ perspectives on receiving genetic information. 1**A** Number of participants initially contacted and the number of participants who returned the feedback form and reported they were already aware of their predisposition or had diagnosis for haemochromatosis. 1**B** Number of participants reporting whether a clinical diagnosis for haemochromatosis had been given to them as a result of this study. 1**C** Increase in the willingness to belong to the Biobank and in trust towards Blood Service as a result of this study reported by the participants. The percentages refer to 1) decreased or partly decreased, 2) neutral and 3) partly grown or grown. 1**D** Participants’ opinions about the usage of genome data in biobanks and receiving genetic information. The percentages refer to 1) totally or partly disagree, 2) neutral and 3) partly or totally agree.

Altogether 94 (0.2%) biobank participants were identified as *HFE* C282Y (+/+) and all these participants had consented for receiving information relevant to their health. Of these participants, 89 had a DNA sample available in the biobank for the confirmatory testing. All the preliminary *HFE* C282Y (+/+) findings, *N* = 89, were confirmed as *HFE* C282Y (+/+) in the external laboratory with an accredited clinical grade method. Due to technical reasons (two participants with missing contact information, five participants with difficulty to interpret the biobank consent for recontacting), 82 biobank participants were contacted in the first phase (Fig. 1A). (Since that, the genetic risk information has been returned to all seven biobank participants but due to the delay in informing, the participants were not administered a survey) Of the 82 biobank participants, 63 returned the feedback form, resulting in response rate of 77%. Of the 63 biobank participants 98% (*N* = 62) were willing to participate in the survey and 100% (*N* = 63) gave a permission to record the finding to blood donation registry. Seven biobank participants, 8.5%, contacted a physician in at The Finnish Red Cross Blood Service after receiving the letter. Of the 62 donors, 6 donors reported in the feedback form they had previous knowledge about their predisposition to haemochromatosis, resulting in 56 donors unaware of their predisposition to haemochromatosis. By combining the information of the 82 donors to the existing self-reported information in the blood donor registry, it was confirmed that, of the 82 biobank participants, only 13.4% (*N* = 11) were aware of their predisposition to haemochromatosis prior this study.

The overall response rate to the survey was 67.7% (*N* = 42), resulting in a response rate of 67.9% (*N* = 38) among biobank participants who weren’t aware of their genetic tendency to haemochromatosis previously (*N* = 56). All survey results, including the clinical results, are self-reported.

Demographic information of the survey participants is available in Table 1. We have previously shown the overall characteristics (age, sex, blood donation activity, blood group, regional differences) of the Blood Service Biobank population compared to the general blood donor population [18]. The sex distribution and the donation activity of the participants reflect well on our previous study of the biobank cohort; female donors represented the majority of the donors participating in the survey and the participants were active and regular blood donors. The donation activity corresponds to the overall high donation activity of the biobank donors [18]: the majority of the respondents, 79% of the females and males, had donated blood at least 21 times. The survey results are shown in more detail in the Supplementary information.

**Table 1:** Demographic characteristics of the *HFE* C282Y (+/+) participating to the survey and not being previously aware of their genetic risk for haemochromatosis. YearStarted refers to the year a participant started donating blood. DonationCount refers to the overall times the participant has donated blood

Characteristic	*N*	Female *N* = 24^*1*^	Male *N* = 14^*1*^
Age	38	52 (43, 62)	50 (42, 60)
YearStarted	34	2000 (1990, 2007)	1997 (1982, 2002)
Unknown		4	0
DonationCount	38		
1–20		5 (21%)	3 (21%)
21–40		11 (46%)	6 (43%)
41–100+		8 (33%)	5 (36%)

^*1*^Median (Q1, Q3); *N* (%)

### Blood donors’ experiences on receiving genetic risk information

Overall, the participants reported positive attitude towards receiving genetic results [23, 24]. Most of the participants found the received information useful (89% strongly agreed and 8% partly agreed). Of the participants, 45% (N=17) felt that receiving genetic information had caused them to worry. In the opinions of the participants’, the genetic information in biobanks should be used more to promote health (76% (N=29) strongly agreed and 24% (N=9) partly agreed) (Figure 1D). Most of the participants (82%) had recognized that consenting to receiving information relevant to health, might lead to receiving health related information. All participants, 100%, wished to receive similar information in the future, should it appear. The participants stated that their trust in The Blood Service and their willingness to belong to The Blood Service Biobank had grown (Figure 1C). All participants, 100%, reported having discussed the finding with their first-degree family members. Based on the open field answers, the participants reported the reactions of the family members to have been mainly neutral with minor concern or confusion. Some of the participants reported having a family history with haemochromatosis in the open fields. The majority of the participants (92%) stated that knowing their genetic risk made them feel motivated to take better care of their health. Eighty-six percent of the participants believe they can affect their predisposition to haemochromatosis with lifestyle choices. Of the participants, 50% (N=19) reported they plan to continue donating blood as before and 34% (N=13) thought they would donate more often in the future than currently. Some participants stated they will no longer donate for another reason, but none of the participants reported stopping future donations because they had received genetic risk information. All but one participant had sought advice from a healthcare provider, as recommended in the letter. It was not clear why one participant did not seek advice from healthcare provider and therefore it cannot be completely ruled out that they were already aware of their genetic predisposition. The participants stated their matter was handled fluently by the healthcare provider (61% (N=23) strongly agreed, 26% (N=19), partly agreed) and that they received enough support from the healthcare provider (strongly agreed 45% (N=17) and partly agreed 45% (N=17)). Most of the participants stated that their questions were answered by their healthcare providers (29% (N=11) strongly agreed, 39% (N=15) partly agreed).

### Self-reported clinical findings

**Fig. 2:**
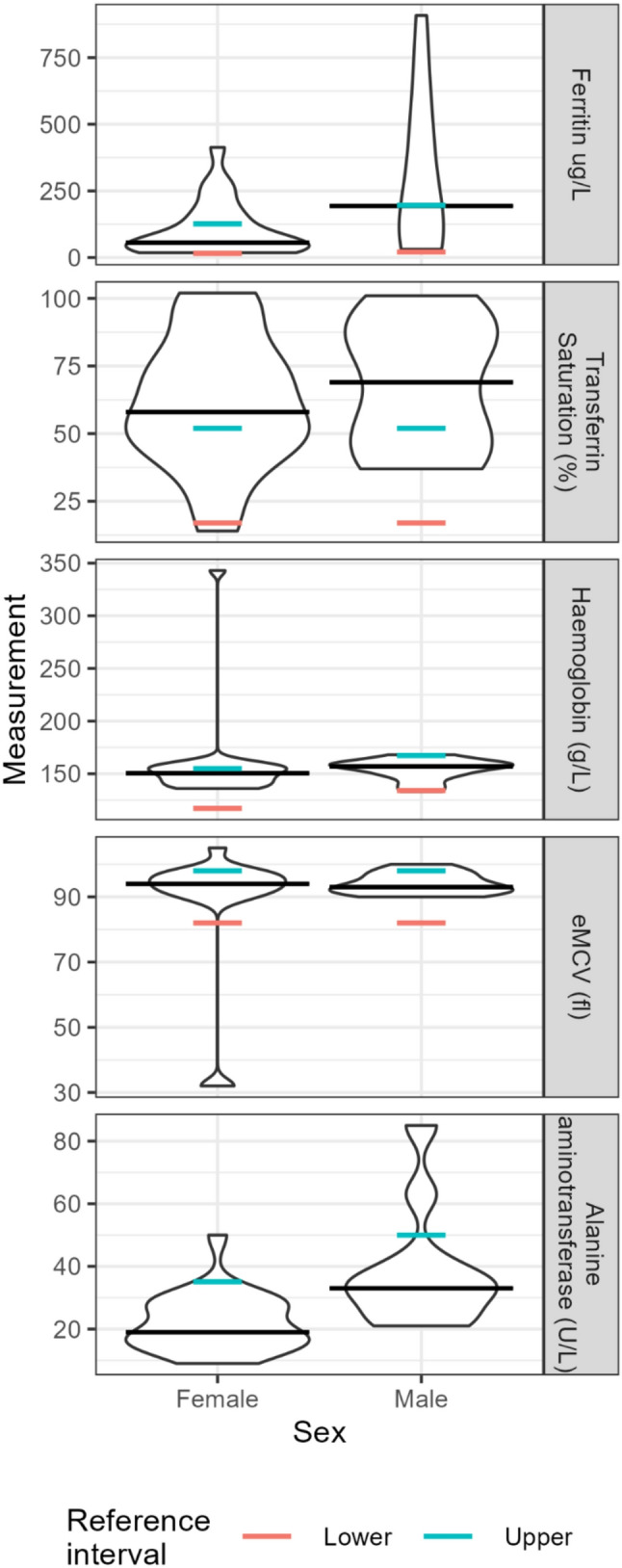
Distribution and median of the clinical laboratory measurements reported by the participants. The unit scale corresponding to each laboratory parameter is depicted on the y-axis. Number of each measurement taken in both sexes is reported in Table 2. Reference intervals are stated in accordance with the reference intervals of the Helsinki University Hospital.

The participants’ self-reported results of the clinical endpoints measured by the healthcare provider are shown in Table 2. The distribution and median of each endpoint are shown in Fig. 2. The number of responses varied between different clinical endpoints and are shown in Table 2. As it is not known where in Finland the survey participants had their clinical measurements taken, all the reference intervals are reported in accordance with the reference intervals of the Helsinki University Hospital. The reference intervals used are shown in detail in the Supplementary information.

Of the participants 63% reported the confirmatory *HFE* C282Y (+/+) from a clinical sample had been taken but putatively because of difficulty in interpreting the laboratory result or insufficient question structure in the survey, the majority of the participants did not report the outcome of the *HFE* C282Y (+/+) test. However, no contradictory findings were reported by the donors or could be observed from the survey results.

The target ferritin level for phlebotomy in the induction phase of haemochromatosis is 50 µg/L and 100 µg/L in the maintenance phase [15]. Accordingly, of the participants, 20.6% reported ferritin levels between 50 µg/L and 99 µg/L, and 47.1% higher than 100 µg/L. Transferrin saturation (TSAT) exceeded the upper bound of reference interval (52%) in 63% of the participants. The alanine aminotransferase (ALT) was mostly within the reference interval (Table 2). High ALT values can indicate liver cell damage [25].

Altogether 36.8% of all the participants reported they had received a clinical diagnosis (ICD10: E83.1) for haemochromatosis. Nine participants reported they didn’t know whether they had received a clinical diagnosis. Of those who responded either yes or no, 56% reported to have received a clinical haemochromatosis diagnosis (Fig. 1B).

**Table 2:** Results of the self-reported clinical laboratory measurements taken in the healthcare. The reference intervals used are those of Helsinki University Hospital. MAD median absolute deviation.

	Female				Male			
**Statistic**	***N***	***Median***	***MAD***	***Reference interval***	***N***	***Median***	***MAD***	***Reference interval***
Haemoglobin (g/L)	22	150	10	117–155	11	157	10	134–167
Ferritin (µg/L)	22	56	40	15–125	12	194	233	20–195
Transferrin Saturation (%)	17	58	21	17–52	10	69	32	17–52
eMCV (fl.)	18	94	4	82–98	11	93	3	82–98
Alanine aminotransferase (U/L)	21	19	9	< 35	11	33	12	< 50

## Discussion

The overall experience of the biobank participants receiving genetic risk information from the Biobank was very positive. From the participants’ perspective the information they received was useful and they’d like to receive similar information in the future, should it appear. However, a need for raising awareness was visible; a portion of the participants reported they didn’t expect such information, and a portion reported they weren’t aware if they had been given a clinical diagnosis (ICD10: E83.1). In the era of genomic medicine, pilot projects like this are an excellent way to increase the genomic information awareness, which is one of the goals of the National Genome Strategy of Finland [3], and has also been endorsed by the European Society of Human Genetics [12]. Another clear aim of this work was to identify blood donors that would benefit from avoiding iron supplementation in future blood donations. Of the participants who were identified with *HFE* C282Y (+/+) genotype, 100% gave the permission to record this finding in the blood donation registry. These blood donors will not be offered iron supplementation in future blood donations.

The self-reported laboratory results indicate relatively high penetrance [26], but based on the self-reported results, no signs of similar severe organ level damage than previously reported [27]. We have previously shown a positive genetic correlation (r_g_ = 0.18) between blood donorship and haemoglobin [28]. It could be possible that individuals with high haemoglobin level are more likely to apply to donate blood and hence, the haemochromatosis prevalence among frequent blood donors is high but regular blood donation has maintained a relatively symptom free state of the haemochromatosis. Also, we have previously shown a high occurrence of ancestral *HLA* haplotype, AH, 7.1 in the Blood Service Biobank [18]. AH 7.1 is known for its association with *HFE* C282Y [29] as well as for its strong protective role in type 1 diabetes, T1D [30, 31]. The high frequency of AH 7.1 in the Blood Service Biobank could be due to blood donation eligibility criteria and the protective role of AH 7.1 in T1D. As clinical manifestations cause blood donation ineligibility, the lower organ level damage in *HFE* C282Y (+/+) can also be caused by selection bias. Therefore, the information about genetic predisposability to haemochromatosis can be of particular importance for those blood donors who no longer fulfill the donation eligibility criteria e.g. due to their age.

To date, *HFE* C282Y (+/+) genotype has been returned to biobank participants e.g. from Geisinger MyCode Community Health Initiative [32]. In this study, a total of 86 601 biobank participants (61,4% females, median (IQR) age was 62.0 (47.0–73.0)) were screened for *HFE* C282Y (+/+) genotype. The number of participants with *HFE* C282Y (+/+) genotype not being aware of their predisposition or diagnosis for haemochromatosis previously was higher in this study (86.6%) than reported in the MyCode study (*N* = 144, 71.6%). The low penetrance of the *HFE* C282Y (+/+) has caused discussion in the past years as to whether the genotype should require action. The biobank scale cohorts enable new possibilities in understanding the impact of common haemochromatosis causing mutations in the clinical outcome of haemochromatosis, which may raise the need for re-evaluation of *HFE* C282Y (+/+) screening at a population level [26]. As a result of this work, we are in the process of screening the entire Blood Service Biobank data set for the *HFE* C282Y (+/+) genotype as well as planning to perform a cost effectiveness analysis to understand the impact of early detected haemochromatosis on healthcare and on individual’s quality of life.

European citizens have in general reported a positive attitude towards biobanking [33, 34]. However, returning genetic information from the biobank is not a routine practice in many countries, including Finland. Maintaining the trust of the biobank participants is of utmost importance. Hence, open, justified and consent-based modes of operation should be prerequisites when genetic information is returned from the biobanks, as well as the existing sufficient intervention and counseling practices. To date, this pilot study is the only one in Finland, where genetic risk information has been returned under the additional consent on willingness to receive information relevant to health given in connection with the biobank consent. The participants’ experience in this work clearly demonstrates the increased trust towards the Finnish Red Cross Blood Service as well as willingness to belong to The Blood Service Biobank as a result of receiving genetic risk information. However, when compared to other actionable genotypes on ACMG [7] or CDC tier 1 [35] list, such as breast cancer related genotypes, *HFE* C282Y (+/+) can be considered a rather “benign” genotype with efficacious interventions. Taken this and the blood donation context into consideration, the broad biobank consent was considered sufficient. It should be noted that this model may not be applicable in all cases, especially in cases with more severe effects of the variant [12]. Moreover, sufficient resourcing for personal counseling when returning genetic information related to more serious conditions, is essential. In this study, few donors contacted a Blood Service physician regarding the results. Although the contacts were positive overall, it emphasizes the importance of receiving personal advice when required.

The key strengths of the study are (1) The timely research topic; returning genetic risk information from the biobank in the transition period of the European Health Data Space, EHDS, regulation (2) A clearly defined research question which benefit in the context of blood donation can be easily and clearly measured; individuals with HFE C282Y (+/+) genotype will not be offered iron supplementation at future donations 3) Demonstrating the value of screening asymptomatic individuals; majority of the participants were not aware of their predisposition or had been diagnosed for haemochromatosis previously 4) The overall high response rate, that improves the reliability of the results and indicates the willingness of the biobank participants to participate in research. Finally, based on the results of this study, a decision was made to screen the rest of the biobank population for HFE C282Y (+/+). However, the study has several limitations. Although the results provide valuable insights on blood donors’ perspective on receiving genetic information, the study is limited by the small number of participants and strictly limited genotype as well as by the self-reported clinical laboratory results. Since the survey study was performed under an informed consent, we could not evaluate the demographic or other type of information of those receiving the *HFE* C282Y (+/+) risk information and responding vs. not responding to the survey. Therefore, despite of the relatively high response rate in the survey, 67.9%, more neutral or even negative experiences cannot be exclusively ruled out. It is possible, that those with positive experience were more willing to take part on the survey. The varying number in respondents reporting the clinical laboratory results could be due to unfinished process in the health care or difficulty to interpret the laboratory results, but however, leads into low number of results and affects the reliability. Furthermore, we have previously reported a high first-degree consanguinity among the biobank blood donors [18]. Although the higher consanguinity can explain the high occurrence of *HFE* C282Y (+/+) genotype in the Blood Service Biobank [18], no direct conclusion can be drawn about the effect of high consanguinity on the observed penetrance.

This work demonstrates how the genetic information stored in biobank can be used in a precisely defined context, such as blood donation. Returning genetic information from biobanks in Finland is currently based on high impact CDC tier1 variants. High impact variants as well as non-malignant results with lower impact, have previously been returned from the Estonian Biobank. The results of this study are consistent with the Estonian study; the majority of the participants in the Estonian Biobank found the received information valuable [36]. Controversiality of the *HFE* C282Y (+/+) genotype, mainly because of the variants’ low penetrance, has caused confusion. In this study we demonstrate a high occurrence of blood donors not being aware of their genetic risk, a relatively high penetrance and a clear acceptance of receiving genetic risk information from the biobank by the participants. Therefore, further comprehensive studies are needed to fully understand the possibilities biobanks may provide in precision-based medicine in the future. Public engagement, nationwide ethical conversation and raising awareness are fundamental steps towards maintaining the public trust and therefore enabling the precision medicine related benefits from biobanks in the future [6].

## Supplementary Information

Supplementary Material 1.

## Data Availability

Aggregate level survey data is available in the article and in the Supplementary information. Analysis scripts are available at https://github.com/FRCBS/hemochromatosis_questionaire.
