# Supplementary material for: Biobank participants’ perspectives on receiving genetic risk information from a biobank – the case of haemochromatosis
Source: BMC Med Genomics. 2025 Dec 17;18:197. doi: 10.1186/s12920-025-02285-3 (PMC12720467; doi:10.1186/s12920-025-02285-3)
Supplement: Supplementary file 1 — Supplementary Material 1. [file 12920_2025_2285_MOESM1_ESM.docx]

Biobank Participants’ Perspectives on Receiving Genetic Risk Information from a Biobank – The case of Haemochromatosis

**SUPPLEMENTARY INFORMATION**

Jonna Clancy^1*^, Janina Forstén^2^, Elina Koskinen^2^, Mikko Arvas^3^, Fredrik Åberg^4^, Kimmo Pitkänen^1#^, Johanna Castrén^2#^

^1^ Finnish Red Cross Blood Service Biobank, Härkälenkki 13, 01730 Vantaa, Finland

^2^ Finnish Red Cross Blood Service, Härkälenkki 13, 01730 Vantaa, Finland

^3^ Finnish Red Cross Blood Service R&D, Biomedicum 1, Haartmaninkatu 8, 00290 Helsinki, Finland

^4^ Transplantation and Liver Surgery, Helsinki University Hospital and University of Helsinki, Helsinki, Finland

^#^ Equal contribution

^*^Corresponding author:

Jonna Clancy, PhD

Finnish Red Cross Blood Service

Biobank/Research & Development

Härkälenkki 13

01730 Vantaa

Finland

+358 50 553 2373

[jonna.clancy@bloodservice.fi](mailto:jonna.clancy@bloodservice.fi)

Contents

[Survey 2](#_Toc198279843)

[Reference intervals 8](#_Toc198279844)

[Survey results 9](#_Toc198279845)

# Survey

SURVEY FOR DONORS WHO HAVE RECEIVED GENETIC RISK INFORMATION – EVALUATION OF THE EXPERIENCE

Research code:________________________

**Background information**

**1. Age**

years

**2. Sex**

- Male
- Female
- Non-binary
- I wish not to answer

**3. How many times have you donated blood?**

In this context, donation refers to the donation of whole blood, not the mechanical donation of plasma or platelets (plasmapheresis or thrombapheresis).

- I have never donated blood
- 1-10
- 11-20
- 21-30
- 31-40
- 51-60
- 61-70
- 71-80
- 81-90
- 91-100
- More than 100

**4. What year did you donate for the first time?**

Please write the year, e.g 2010

**5. How are you planning to continue blood donation after receiving the information about iron accumulation tendency?**

- More often than nowadays
- Like nowadays
- More seldom than nowadays
- I am going to stop blood donation because of the information I’ve received
- I am going to stop blood donation for some other reason, eg. my age
- I can’t tell

**6. The received genetic information was easy to understand**

- Yes
- No
- I don’t know

**7. Did you understand when you gave the biobank consent that it could lead to receiving information relevant to health?**

- Yes
- No

**8. Would you like to receive similar information in the future, should it appear?**

- Yes
- No

If you wish, you can complete your answer

Evaluate the following statements below based on your experience

**9. It was useful to receive genetic information**

- Fully agree
- Partly agree
- I don’t disagree or agree
- Party disagree
- Fully disagree

**10. Receiving genetic information did not worry me**

- Fully agree
- Partly agree
- I don’t disagree or agree
- Party disagree
- Fully disagree

**11. The genome data in biobanks should be used more widely to promote health**

- Fully agree
- Partly agree
- I don’t disagree or agree
- Party disagree
- Fully disagree

**12. How has receiving genetic information affected your trust in The Blood Service?**

- Has strengthened
- Has partly strengthened
- Has had no effect
- Has partly weakened
- Has weakened

If you wish you can complete your answer

**13. How has receiving genetic information affected to your willingness to belong to the Blood Service Biobank?**

- Has strengthened
- Has partly strengthened
- Has had no effect
- Has partly weakened
- Has weakened

**14. After receiving the information, did you apply for health care?**

- Yes
- No

If you answered “No” to question 14, you can continue from question 23.

**15. To which health care facility did you apply?**

- Student health care
- Occupational health
- Specialized medical care
- Public health care
- Private health care
- Other, which____________

Evaluate the statements below according to your experience

**16. My situation was taken care of fluently in healthcare**

- Fully agree
- Partly agree
- I don’t disagree or agree
- Party disagree
- Fully disagree

**17. I got enough support from health care**

- Fully agree
- Partly agree
- I don’t disagree or agree
- Party disagree
- Fully disagree

**18. My questions were answered in health care**

- Fully agree
- Partly agree
- I don’t disagree or agree
- Party disagree
- Fully disagree

**19. In health care services, the attitude towards the returned information from Biobank was…**

- Positive
- Partly positive
- Neutral
- Partly negative
- Negative

**20. Were you subjected to the following laboratory tests in healthcare and what were their results?**

The test was taken Result

Yes No

Confirmation of HFE C282Y +/+ 🞎 🞎

Ferritin 🞎 🞎 µ/l

Transferrin saturation %

Basic blood count, hemoglobin g/l

Basic blood count, MCV fl

Alanine aminotransferase U/l

*Note: these laboratory tests were referred here in the same manner as they were originally referred in the letter where the participants were guided to apply health care and have these laboratory tests done.*

**21. Did you undergo any other tests? If yes, what tests and what were the results?**

**22. Did you get a clinical diagnosis for hemochromatosis (ICD10: E83.1)?**

- Yes
- No
- I don’t know

**23. Have you had the following symptoms or diagnosed diseases?**

Tiredness 🞎 Yes 🞎 No

Joint symptoms 🞎 Yes 🞎 No

Type 2 diabetes 🞎 Yes 🞎 No

Some other symptoms, what?

Evaluate the statements below

**24. I believe that I can influence my risk of hemochromatosis with my lifestyle**

- Fully agree
- Partly agree
- I don’t disagree or agree
- Party disagree
- Fully disagree

**25. Receiving risk information motivates me to take better care of my health**

- Fully agree
- Partly agree
- I don’t disagree or agree
- Party disagree
- Fully disagree

**26. I have discussed about my tendency to haemochromatosis with my close relatives**

Close relative means biological parents, siblings and children

- Yes
- No

If yes, with how many?

If you wish, you can tell how the information was received among the relatives or what kind of thoughts may have been brought up

We appreciate your time regarding the research

# Reference intervals

The reference intervals are reported in accordance with Helsinki University Hospital reference: https://diagnostiikka.hus.fi/hakusivu?search=&tabs=ebs

| **Laboratory measurement** | **Female** | **Male** |
| --- | --- | --- |
| P -Ferrit µg/l | 15 - 125 | 20 - 195 |
| fP-Trfesat % | 17 - 52 | 17 - 52 |
| Basic blood count, haemoglobin g/L | 117 - 155 | 134 - 167 |
| Basic blood count, MCV fl | 82 - 98 | 82 - 98 |
| Alanine transaminase U/l | < 35 | < 50 |

# Survey results

**Background information**

**1. Age**

**2. Sex**

**3. How many times have you donated blood?**

**4. What year did you donate for the first time?**

*Results for the questions 1-4 are shown in the article Table 1*

**5. How are you planning to continue blood donation after receiving the information about iron accumulation tendency?**

**
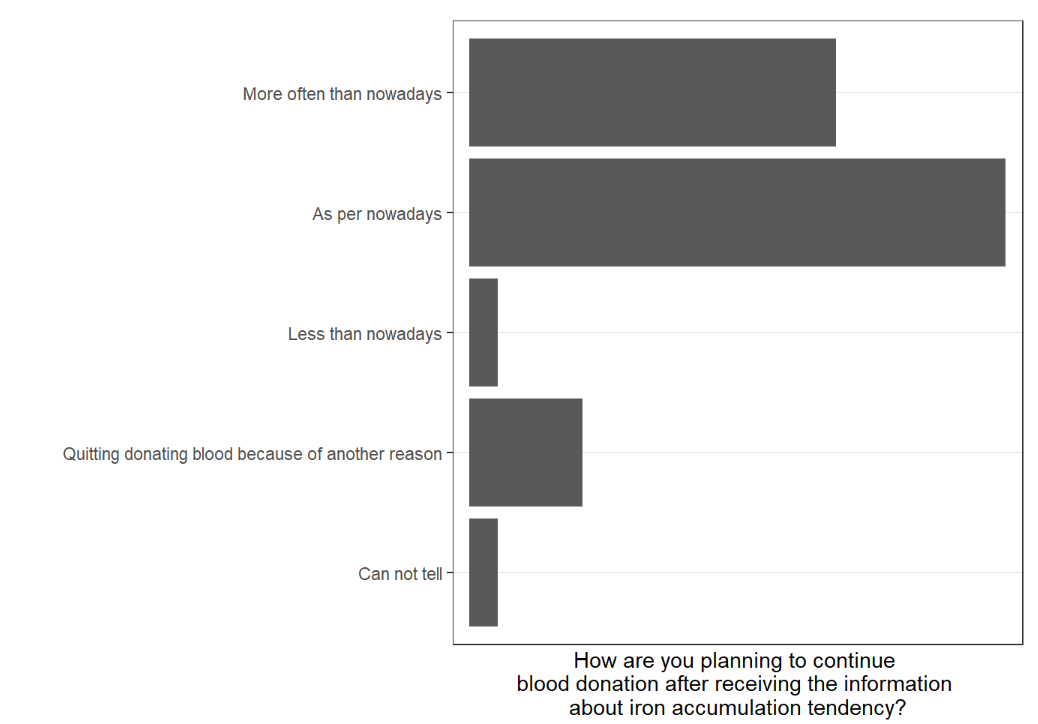
**

**6. The received genetic information was easy to understand**


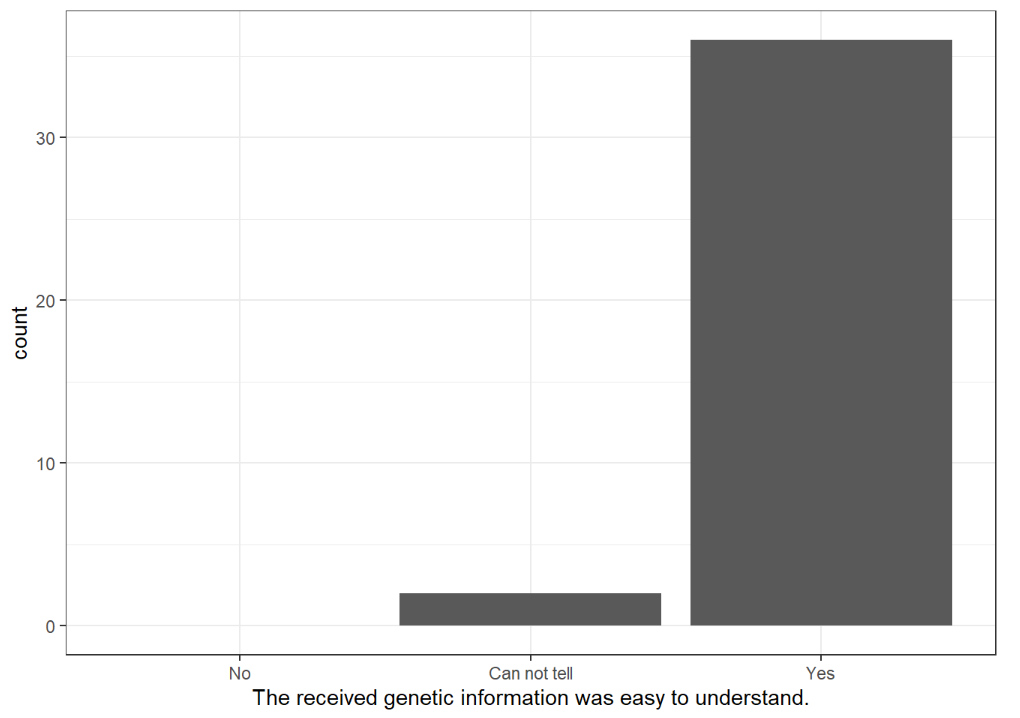


**7. When you gave your consent for biobank, did you understand it could lead to receiving information relevant to your health?**

**
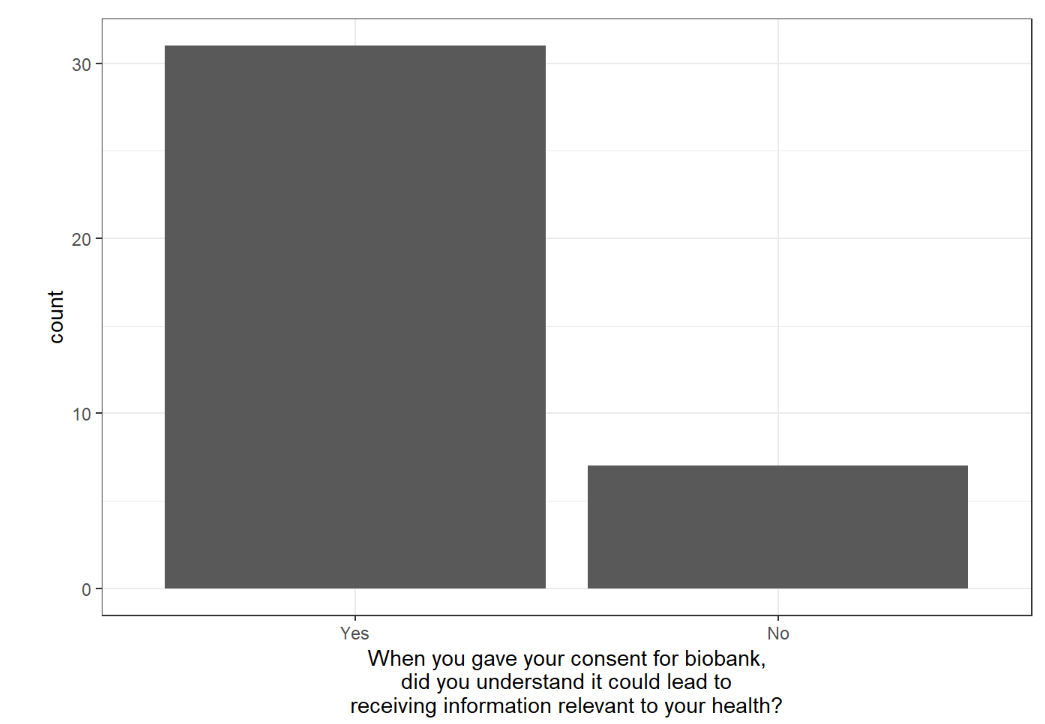
**

**8. Would you like to receive similar information in the future, should it appear?**


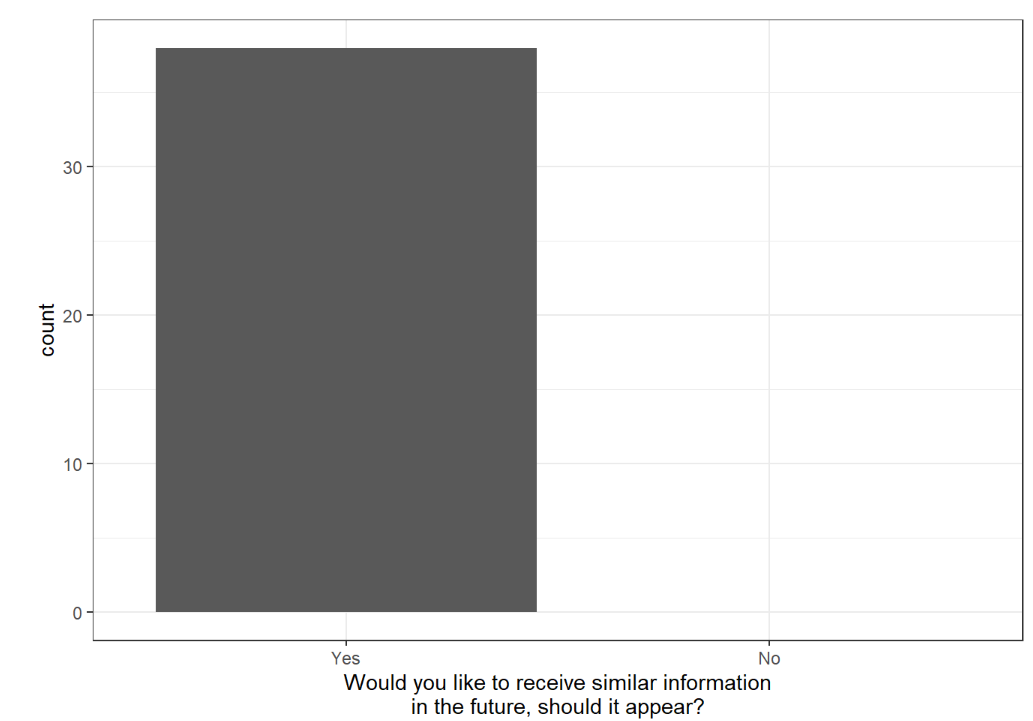


**9. It was useful to receive genetic information**

**10. Receiving genetic information did not worry me**

**11. The genome data in biobanks should be used more widely to promote health**

*Results for the questions 9-11 are shown in the article Figure 1D*

**12.** **How has receiving genetic information affected your trust in The Blood Service?**

**13. How has receiving genetic information affected to your willingness to belong to the Blood Service Biobank?**

*Results for the questions 12-13 are shown in the article Figure 1C*

**14. After receiving the information, did you apply for health care?**


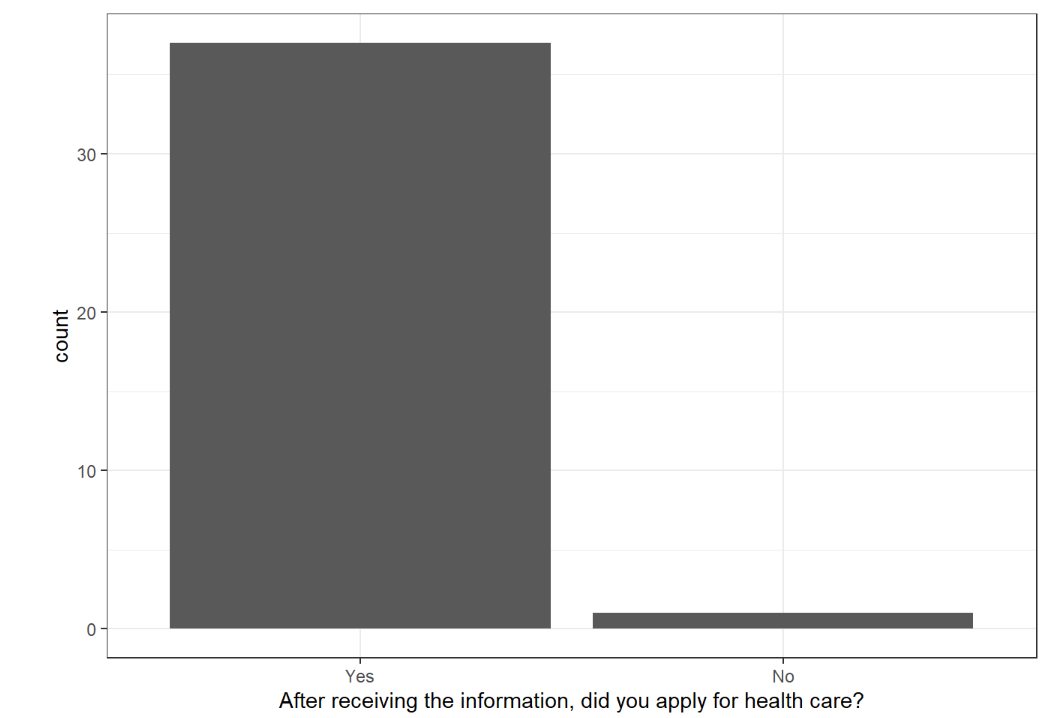


**15. To which health care facility did you apply?**

**
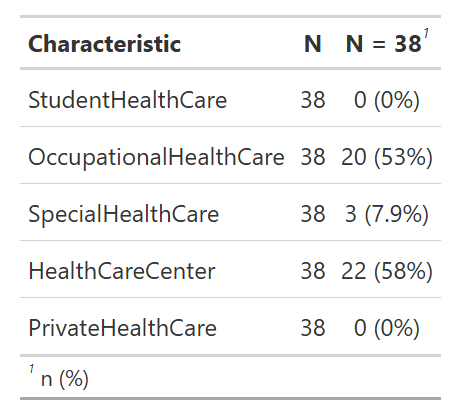
**

**16. I got enough support from health care**

**17. My situation was taken care of fluently in healthcare**

**18. My questions were answered in health care**

**
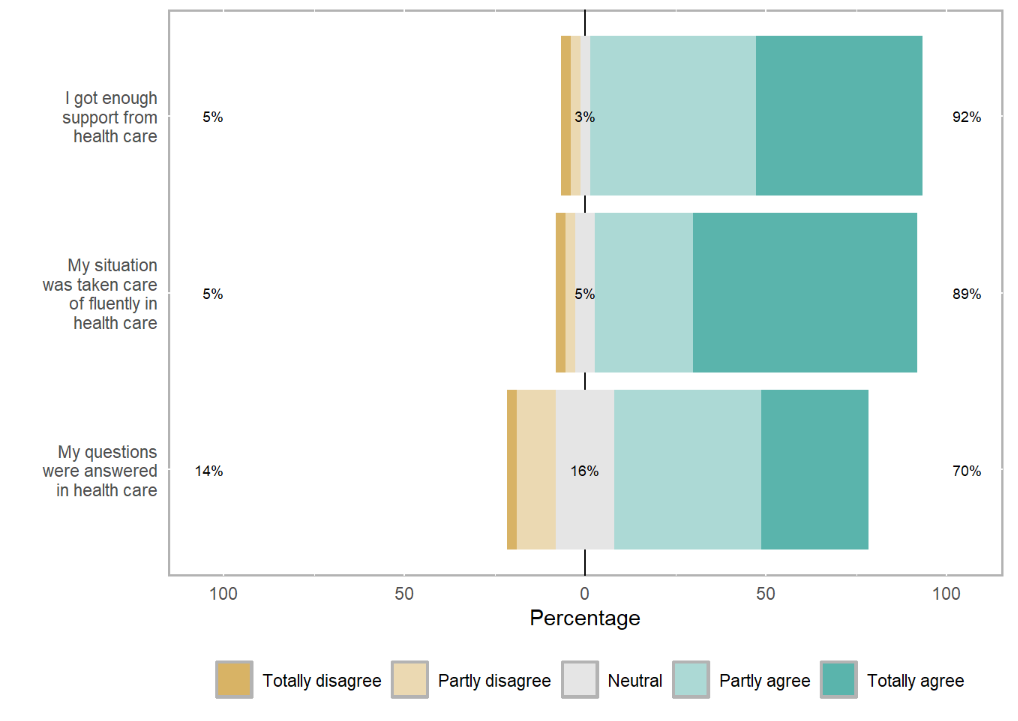
**

**19. In health care services, the attitude towards the returned information from Biobank was…**


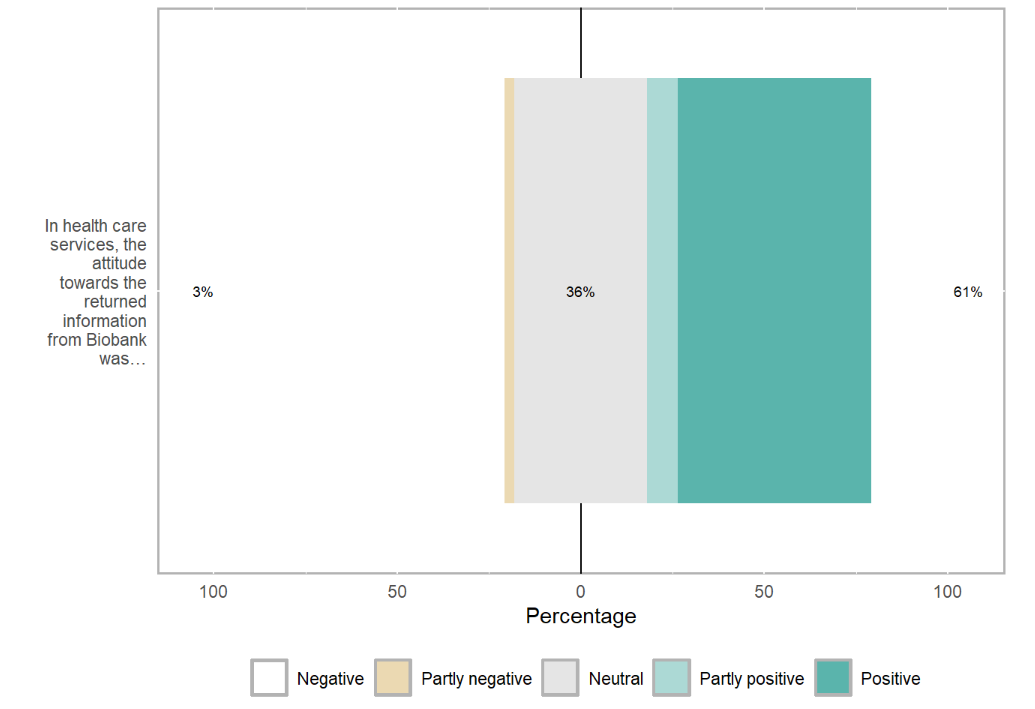


**20. Were you subjected to the following laboratory tests in healthcare and what were their results?**

*Results for the question 20 are shown in the article Table 2*

**21. Did you undergo any other tests? If yes, what tests and what were the results?**

*Open field answers are not shown*

**22. Did you get a clinical diagnosis for hemochromatosis (ICD10: E83.1)?**

**
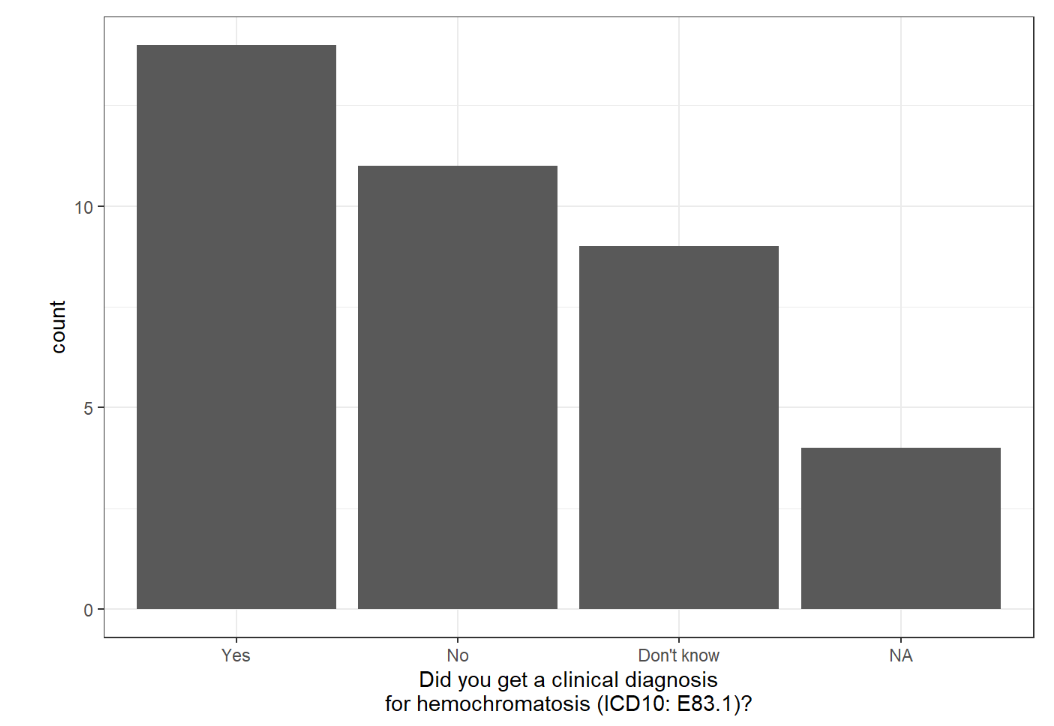
**

**23. Have you had the following symptoms or diagnosed diseases?**


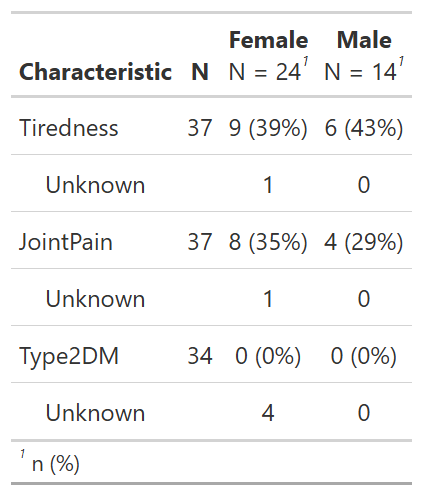


< 5

**24. I believe I can make an impact to my predisposition to haemochromatosis with my lifestyle**

**25. Receiving genetic risk information motivates me to take care of my health better**

*Results for the questions 24-25 are shown in the article Figure 1D*

**26. I have discussed about my tendency to hemochromatosis with my close relatives**

If yes, with how many?

*Nearly all the participants reported they had discussed about their tendency with their close relatives.*
